# *Staphylococcus aureus *enterotoxins induce IL-8 secretion by human nasal epithelial cells

**DOI:** 10.1186/1465-9921-7-115

**Published:** 2006-09-04

**Authors:** Garrett J O'Brien, Gareth Riddell, J Stuart Elborn, Madeleine Ennis, Grzegorz Skibinski

**Affiliations:** 1 Respiratory Research Group, School of Medicine and Dentistry, Queen's University Belfast, Grosvenor Road, Belfast BT12 6BJ, Northern Ireland, UK

## Abstract

**Background:**

*Staphylococcus aureus *produces a set of proteins which act both as superantigens and toxins. Although their mode of action as superantigens is well understood, little is known about their effects on airway epithelial cells.

**Methods:**

To investigate this problem, primary nasal epithelial cells derived from normal and asthmatic subjects were stimulated with staphylococcal enterotoxin A and B (SEA and SEB) and secreted (supernatants) and cell-associated (cell lysates) IL-8, TNF-α, RANTES and eotaxin were determined by specific ELISAs.

**Results:**

Non-toxic concentrations of SEA and SEB (0.01 μg/ml and 1.0 μg/ml) induced IL-8 secretion after 24 h of culture. Pre-treatment of the cells with IFN-γ (50 IU/ml) resulted in a further increase of IL-8 secretion. In cells from healthy donors pretreated with IFN-γ, SEA at 1.0 μg/ml induced release of 1009 pg/ml IL-8 (733.0–1216 pg/ml, median (range)) while in cells from asthmatic donors the same treatment induced significantly higher IL-8 secretion – 1550 pg/ml (1168.0–2000.0 pg/ml p = 0.04). Normal cells pre-treated with IFN-γ and then cultured with SEB at 1.0 μg/ml released 904.6 pg/ml IL-8 (666.5–1169.0 pg/ml). Cells from asthmatics treated in the same way produced significantly higher amounts of IL-8 – 1665.0 pg/ml (1168.0–2000.0 pg/ml, p = 0.01). Blocking antibodies to MHC class II molecules added to cultures stimulated with SEA and SEB, reduced IL-8 secretion by about 40% in IFN-γ unstimulated cultures and 75% in IFN-γ stimulated cultures. No secretion of TNF-α, RANTES and eotaxin was noted.

**Conclusion:**

Staphylococcal enterotoxins may have a role in the pathogenesis of asthma.

## Background

*Staphylococcus aureus *(*S. aureus*) is a common human pathogen associated with various local and systemic infections, characterized by inflammation dominated by polymorphonuclear leukocytes. It produces a set of toxins including staphylococcal enterotoxins and toxic shock syndrome toxin-1 which cause food poisoning and toxic shock syndrome respectively in humans and other species. These toxins are intermediate molecular weight proteins (22-20 kD) that also act as superantigens (SAgs) due to their ability to bind to MHC class II molecules on antigen presenting cells and stimulate all T cells bearing particular V βs on their T cell receptors [1].

The epithelium acts as a physiological barrier to diffusion [2] and after physical or chemical damage has occurred, inhaled allergens, irritants and agonists can have detrimental effects on the underlying smooth muscle [3]. Traditionally, the epithelium was considered to be an inert barrier dividing the external environment and the inner tissue of the lung. However, it is now accepted that it constitutes the interface between the internal milieu and the external environment and plays a pivotal role in controlling many airway functions including barrier and secretory functions [4-6]. Airway hyper-responsiveness and epithelial cell damage are associated commonly with asthma.

In view of the ever increasing evidence for the effects of staphylococcal superantigens on immuno-modulatory and pro-inflammatory cells, it is likely that there is an association between staphylococcal infection and the pathogenesis of atopic diseases such as dermatitis, rhinitis and asthma [7,8]. Enterotoxins produced by *S. aureus *and their specific IgE antibodies are thought to be important in worsening atopic dermatitis [7].

Studies have shown greater *S. aureus *colonisation in the skin of patients with atopic eczema/dermatitis syndrome (AEDS) (80–100%) than in the skin of normal healthy subjects (5–30%). Indeed *S. aureus *constitutes up to 80% of the normal flora in atopic individuals and *S. aureus *isolated from the skin of at least 65% of AEDS patients secretes the Sags, *S. aureus *enterotoxin A (SEA), *S. aureus *enterotoxin B (SEB), *S. aureus *enterotoxin C (SEC), *S. aureus *enterotoxin D (SED) and Toxic Shock Syndrome Toxin-1 (TSST-1) [9].

In humans it is the nasal passage which is the most common site for *S. aureus *colonization [10]. Whereas more than 50 % pathogenic isolates of *S. aureus *produce one or more SAgs exotoxins, even strains isolated from asymptomatic carriers can produce SAgs [11]. Given their anatomical localization and ability to produce exotoxins, it is likely that the nasal passage is exposed to bacterial SAgs [1].

In comparison to AEDS, few studies have documented the role of *S. aureus *or its SAgs in allergic or non-allergic airway disease. Earlier investigations suggested an allergy to certain bacteria as an important cause of exacerbation of the disease in patients suffering from allergic airway disease [12,13]. However, the tests used whole bacterial lysates, were highly unspecific and no correlations were found among these results.

Interferon-gamma (IFN-γ) is known to induce major histocompatibility complex class II expression on bronchial epithelial cells *in vitro *[14,15]. *In vivo *the expression of MHC class II molecules is enhanced in asthma and lung neoplastic disease, allowing bronchial epithelial cells to function as antigen presenting cells and to interact with T cells [15,16]. Although the major role of MHC class II is to present antigens to T cells, engagement of MHC class II by superantigens and other bacterial products has also consequences for the class II expressing cells including increased cytokine secretion and apoptosis [16-18].

Even though the MHC class II molecule appears to be the major receptor for the staphylococcal enterotoxins, it has been shown that antibodies to major histocompatibility complex I (MHC class I) can inhibit the binding of SEA and SEB to MHC class II negative macrophages [19]. Studies performed with MHC class II negative epithelial cell line demonstrated modulation of intracellular Ca^2+ ^signal pathway in response to SEA [20]. These findings suggest that MHC class II molecule may not be the only receptor for staphylococcal exotoxins.

It has been recently demonstrated that interaction of live *S. aureus *with human tracheal epithelial cell line MM-39 stimulates release of IL-8, eotaxin and RANTES [21]. Our study investigates the effect of *S. aureus *products, SEA and SEB, on human nasal epithelial cells and tests the hypothesis that SEA and SEB can induce the release of proinflammatory cytokines from human nasal epithelial cells.

## Materials and methods

Subjects were recruited from staff and students at Queen's University Belfast or the Belfast City Hospital. The study was approved by the Research Ethics Committee of Queen's University Belfast and all participants provided written informed consent. All subjects were non-smokers and were between 22–39 years old. They were in good general health and had no history of cardiac or renal disease.

Control subjects had no history of respiratory symptoms and some were atopic. Asthmatic subjects had a clinical history of physician-diagnosed asthma, with intermittent shortness of breath or wheeze within the previous 12 months. All subjects had an FEV_1 _of at least 60% predicted. They were not taking regular anti-inflammatory therapy and were maintained only on short-acting β_2 _agonists. No subject had previously been prescribed a long acting β_2 _agonist. They had not taken either inhaled or oral steroids in the six months preceding the commencement of the study. They had been free from upper respiratory tract infections for a minimum of four weeks preceding the commencement of the study. Atopy was defined by positive skin prick tests to 1 or more of 4 common environmental allergens, including house dust mite (*Dermatophagoides pterynonisinus *(HDM), mixed grass pollen, cat and dog hair. Standardised allergen preparations (Dome-Hollister-Stier, Epernon Cedex, France) of house dust mite (*Dermatophagoides pterynonisinus*) (HDM), mixed grass pollen, cat and dog hair were applied to the volar aspect of the forearm, using a standard puncture technique as described by the European Academy of Allergology and Clinical Immunology [22]. Standardised solutions of histamine (1% w/v) and saline were used as positive and negative controls respectively. Atopy was defined as having one or more positive skin prick tests to test allergen solutions.

### Spirometry

Spirometry was performed on all subjects. Spirometry was performed according to the American Thoracic Society Guidelines using a Vitalograph spirometer [23]. Prior to attending for spirometry, subjects were asked to withhold short acting β_2 _acting agonists for at least eight hours. Records were taken of the subjects' height, weight and age. Predicted values for spirometry were then calculated from validated equations [24].

### Isolation of primary human nasal epithelial cells

Nasal brushings were performed on all subjects using a standardized protocol and no local anaesthetics were used during the procedure. A bronchial cytology brush (TeleMed Systems Inc., MA, USA) was used to obtain two brushings from the external turbinate of each nostril. Each nostril was brushed once and the process was repeated providing the subject tolerated the process.

Cells were cultured in BEGM medium (Clonetics) until passage. Cells from passage 1 were frozen in liquid nitrogen and stored until used in experiments at passage 2–3. All the cells used in this work stained positive with pan-cytokeratine, cytokeratine 5+8, cytokeratin 8, cytokeratin 18 and negative with anti-vimentin and anti-cytokeratin 13 (not shown). Cells were grown in submersion cultures. For the experiments, human nasal epithelial cells (HNECs) were seeded into 24 well plates using a seeding density of 2 × 10^5 ^cells/ml and a well volume of 300 μl. Cells were incubated at 37°C, 5% CO_2 _for 6 h. After the 6 h incubation period the cells were washed with PBS (37°C, pH 7.4) and fresh BEGM with or without 50 IU/ml IFN-γ was added to the wells. Cells were left to incubate at 37°C for a further 24 h. After 24 h (cells 80–90% confluent), media was removed and the cells were washed with PBS (37°C, pH 7.4) and fresh media added containing either SEA or SEB (0.01 and 1 μg/ml) or nothing (control). Supernatants were collected 6 and 24 h post stimulation and stored at -80°C until analysed by ELISA. In selected experiments before enterotoxin stimulation blocking anti-MHC class II antibody (IgG2a, clone L243, BioLegend, San Diego, CA) was added at 50 μg/ml for 1 hour to cell cultures. After incubation enterotoxins were added as described above. The concentration of antibody used inhibited detection of MHC class II on human monocytic THP-1 cell line by 95%. Purified mouse IgG2a (MOPC-173, BioLegend) was used as control.

### Spiking experiments

Nasal epithelial cells were seeded into 24-well plates using a density of 2.5 × 10^5 ^cells/ml and a well volume of 300 μl. Cells were stimulated with either 1 μg/ml SEA or SEB. The supernatant was collected at 24 h of culture. Spiking was carried out by splitting the supernatant into two aliquots. The first aliquot was spiked with 500 pg/ml of either TNF-α, RANTES or eotaxin. Cytokine concentrations were then measured by ELISA in both portions.

### Cell lysate experiments

Once supernatants were collected, fresh BEGM (300 μl) was added to each well. To lyse the cells the 24-well plate was freeze-thawed three times. The lysate was then centrifuged at 300 *g *for 5 min and subsequently aliquoted. Cytokine concentrations in lysates were measured by ELISA.

### ELISA assay

Cytokine analyses were carried out using sandwich ELISA according to manufacturer's instructions (R & D Systems).

### Reagents

Recombinant IFN-γ was purchased from PeproTech EC (London, UK). SEA and SEB were purchased from Sigma-Aldrich (Poole, UK). SEA and SEB were used in concentrations of 0.01 and 1 μg/ml which in preliminary experiments have been shown to be non-toxic for epithelial cells by MTT and trypan blue exclusion tests.

### Flow cytometric analysis

Nasal epithelial cells were detached from culture dishes by means of nonenzymatic cell dissociation solution (Sigma) and were then stained with anti-HLA-DR, P, Q FITC conjugated monoclonal antibody (DAKO). MHC class II expression epithelial cells was assessed by flow cytometry (EPICS II; Coulter, Hialeah, Fl). Results were expressed as % of positive cells and as mean fluorescence intensity.

### Statistical analysis

Results are reported as median (range). Statistical comparisons were performed using Mann-Whitney U test, Friedman (Dunn's post-hoc test) and Wilcoxon matched pair test. All statistical analyses were carried out using SPSS (Version 11.5) for Windows and GraphPadPrism^®^. GraphPadPrism^® ^was used to plot graphs.

## Results

### Subject characteristics

A total of 20 subjects were included in the study (mean age 26.95 ± 4.19 y, 10 female). Subject characteristics are summarised in table 1.

**Table 1:** Subject Demographics

**Patient ID**	**Age (years)**	**Sex**	**Status**	**Atopy**	**FEV_1 _(L)**	**FEV_1 _% pred**	**FVC (L)**
1	34	M	Normal	NA	4.5	110	6.2
3	25	M	Normal	A	4.6	108	5.7
4	27	M	Normal	NA	4.0	92	5.3
5	24	F	Normal	NA	3.4	103	3.6
6	25	M	Normal	NA	5.2	122	6.35
8	39	F	Normal	NA	2.6	116	3.3
9	25	F	Normal	A	3.6	100	4.0
10	23	F	Normal	A	3.2	98	3.3
14	27	M	Normal	NA	5.2	111	6.4
17	27	F	Normal	NA	3.1	98	4.1
2	32	M	Asthmatic	A	4.9	121	4.7
7	32	M	Asthma	A	4.5	82	6.4
11	27	M	Asthmatic	A	3.1	87	4.1
12	25	F	Asthmatic	A	3.1	97	3.7
13	24	F	Asthmatic	A	3.4	114	3.8
15	27	M	Asthmatic	NA	3.9	95	5.4
16	22	F	Asthmatic	A	2.9	102	3.6
18	25	M	Asthmatic	A	3.3	79	4.7
19	24	F	Asthmatic	A	2.9	94	3.4
20	25	F	Asthmatic	A	3.7	104	4.2

M = male, F = female; A = atopic, NA = non-atopic; FEV_1 _= forced expiratory volume in one second; FVC = forced vital capacity; L = litres.

### Effect of Enterotoxin A (SEA) and Enterotoxin B (SEB) on primary human nasal epithelial cells

#### Basal release of IL-8

**Figure 1:**
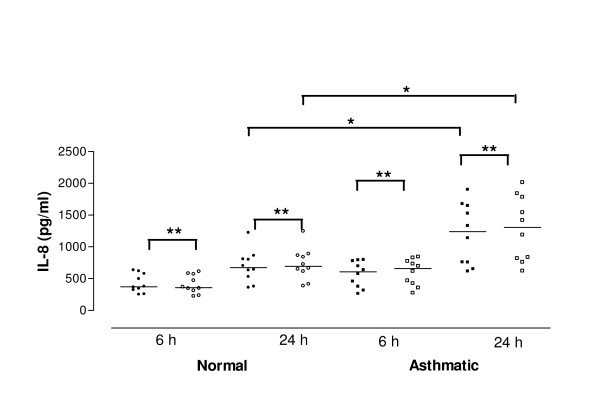
Release of interleukin-8 (IL-8) in cell culture supernatants after interferon-gamma (IFN-γ) pretreatment. Data are shown as individual points, the line represents the median. Black symbols = no IFN-γ pretreatment, open symbols = IFN-γ pretreatment. P values reaching statistical significance are marked on the graph. * = P < 0.05; ** = P < 0.01.

There was no significant difference in the baseline release of IL-8 from non-stimulated cells derived from normal (358.4 pg/ml, 304.2–509.6 pg/ml) and asthmatic subjects (607.9 pg/ml, 424.9–717.4 pg/ml, p = 0.06) at 6 h. However, in contrast cells derived from asthmatic subjects released significantly more IL-8 at 24 h compared to those derived from control subjects (normal control 671.8 pg/ml, 511.8–875.0 pg/ml; asthmatic 1239.0 pg/ml, 859.9–1547 pg/ml, p = 0.03) (Figure 1).

#### Effect of IFN-γ on IL-8 release

IFN-γ pre-treatment (50 U/ml) of cells derived from normal subjects increased baseline IL-8 release significantly at 6 h (370.4 pg/ml, 320.6–528.6 pg/ml, p < 0.01) and 24 h (694.4 pg/ml, 549.5–908.7 pg/ml, p < 0.01). Similarly, IL-8 release from cells derived from asthmatic subjects was increased significantly at 6 h (657.7 pg/ml, 453.5–749.0 pg/ml, p < 0.01) and 24 h (1304.0 pg/ml, 919.6–1645.0 pg/ml, p < 0.01) (Figure 1). Although there was no significant difference in the baseline release of IL-8 after IFN-γ pre-treatment between cells derived from normal and asthmatic subjects at 6 h (p > 0.05) the difference was significant at 24 h (p = 0.02).

#### IL-8 release in response to SEA

**Figure 2:**
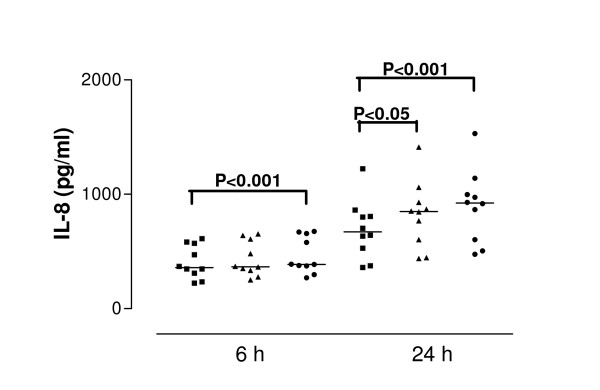
Interleukin-8 (IL-8) release from cells derived from normal subjects in response to SEA after 6 and 24 hour stimulation. Data are shown as individual points, the line represents the median. ■ = control (unstimulated cells), ▲ = 0.01 μg/ml SEA, ● = 1 μg/ml SEA. Median values of IL-8 release at 6 hours: control P values reaching statistical significance are indicated on the graph.

**Figure 3:**
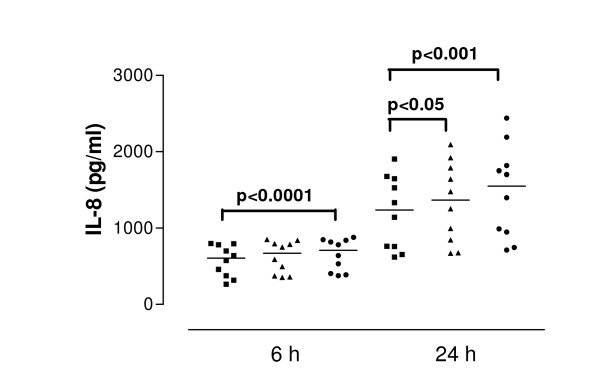
Interleukin-8 (IL-8) from cells derived from asthmatic subjects in response to SEA after 6 and 24 hour stimulation. Data are shown as individual points, the line represents the median. ■ = control (unstimulated cells), ▲ = 0.01 μg/ml SEA, ● = 1 μg/ml SEA. P values reaching statistical significance are indicated on the graph.

**Figure 4:**
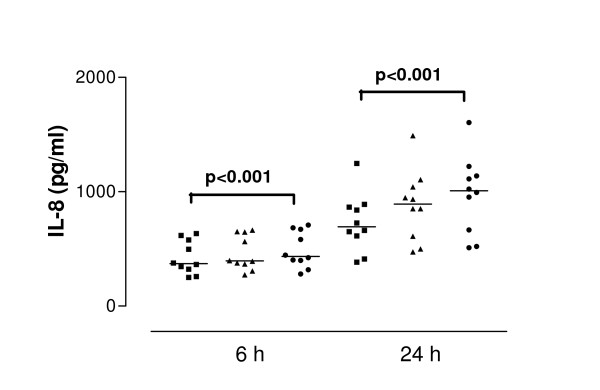
Interleukin-8 (IL-8) release from interferon-gamma (IFN-γ)-treated cells derived from normal subjects in response to SEA after 6 and 24 hour stimulation. Data are shown as individual points, the line represents the median. ■ = control (unstimulated cells), ▲ = 0.01 μg/ml SEA, ● = 1 μg/ml SEA. P values reaching statistical significance are indicated on the graph.

**Figure 5:**
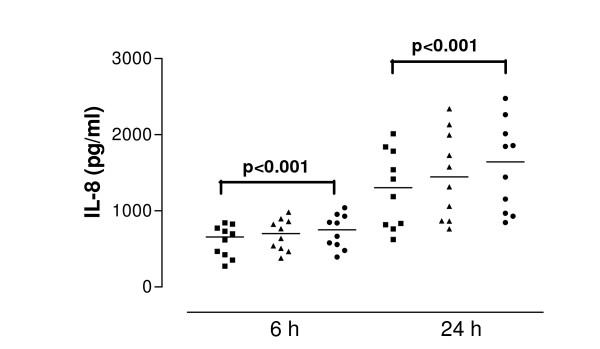
Interleukin-8 (IL-8) release from interferon-gamma (IFN-γ)-treated cells derived from asthmatic subjects in response to SEA after 6 and 24 hour stimulation. Data are shown as individual points, the line represents the median. ■ = control (unstimulated cells), ▲ = 0.01 μg/ml SEA, ● = 1 μg/ml SEA. P values reaching statistical significance are indicated on the graph.

SEA caused significant IL-8 release from nasal epithelial cells derived from control and asthmatic subjects at both 6 and 24 h (both concentrations tested). The median values of IL-8 concentration were at 6 h incubation with 1.0 μg/ml SEA – 387.0 pg/ml, at 24 h incubation 848.8 pg/ml for 0.01 μg/ml SEA and 923.7 pg/ml for 1.0 μg/ml SEA. Cells derived from asthmatic subjects released significantly more IL-8 than those from control subjects at both 6 h (p = 0.04) and 24 h (p = 0.02) (Figure 2 and 3). The median value of IL-8 release for 6 hour stimulation with 0.01 μg/ml SEA was 710.9 pg/ml: for 24 hour stimulation with 0.01 μg/ml SEA – 1035 pg/ml and with 1.0 μg/ml – 1367 pg/ml. Pretreatment of cells with IFN-γ (50 U/ml) followed by toxin stimulation resulted in increased IL-8 release. In cells from healthy donors pretreated with IFN-γ, SEA at 1.0 μg/ml induced release of 1009 pg/ml IL-8 (median value) while in cells from asthmatic donors the same treatment induced significantly higher IL-8 secretion – 1550 pg/ml (p = 0.04). (Figure 4 and 5).

#### IL-8 release in response to SEB

**Figure 6:**
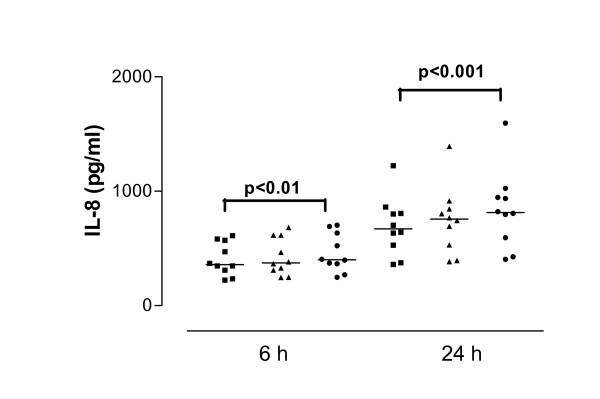
Interleukin-8 (IL-8) release from cells derived from normal subjects in response to SEB after 6 and 24 hour stimulation. Data are shown as individual points, the line represents the median. ■ = control (unstimulated cells), ▲ = 0.01 μg/ml SEB, ● = 1 μg/ml SEB. P values reaching statistical significance are indicated on the graph.

**Figure 7:**
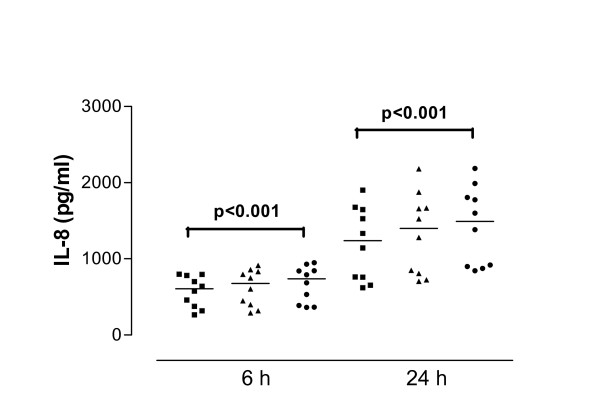
Interleukin-8 (IL-8) release from cells derived from asthmatic subjects in response to SEB after 6 and 24 hour stimulation. Data are shown as individual points, the line represents the median. ■ = control (unstimulated cells), ▲ = 0.01 μg/ml SEB, ● = 1 μg/ml SEB. P values reaching statistical significance are indicated on the graph.

**Figure 8:**
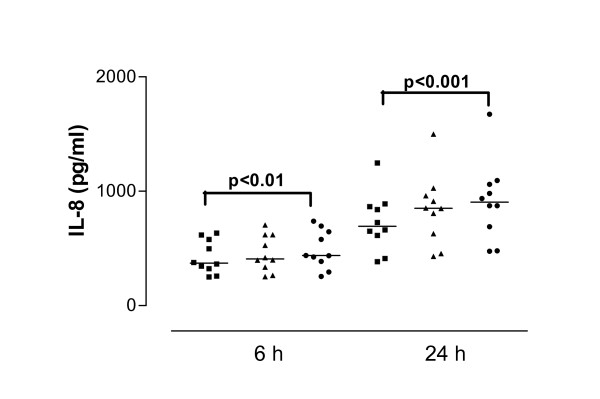
Interleukin-8 (IL-8) releases from interferon-gamma (IFN-γ)-treated cells derived from normal subjects in response to SEB after 6 and 24 hour stimulation. Data are shown as individual points, the line represents the median. ■ = control (unstimulated cells), ▲ = 0.01 μg/ml SEB, ● = 1 μg/ml SEB. P values reaching statistical significance are indicated on the graph.

**Figure 9:**
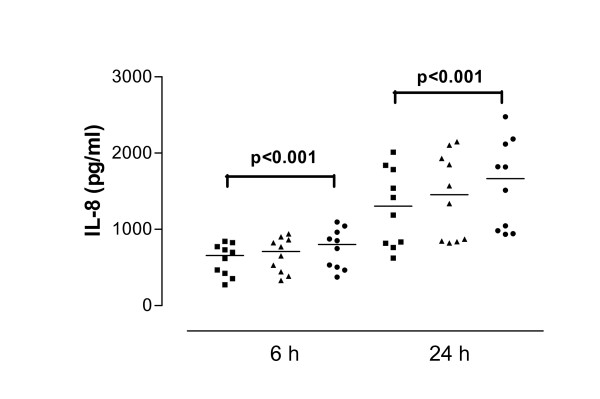
Interleukin-8 (IL-8) release from interferon-gamma (IFN-γ)-treated cells derived from asthmatic subjects in response to SEB after 6 and 24 hours stimulation. Data are shown as individual points, the line represents the median. ■ = control (unstimulated cells), ▲ = 0.01 μg/ml SEB, ● = 1 μg/ml SEB. P values reaching statistical significance are indicated on the graph.

The highest concentration of SEB tested (1 μg/ml) induced significant IL-8 release from cells derived from normal and asthmatic subjects at both 6 and 24 h. The median values of IL-8 release were 400.8 pg/ml for 6 hour stimulation and 814.0 pg/ml for 24 hour stimulation. SEB (1 μg/ml) induced significantly more IL-8 release from cells derived from asthmatic subjects compared to cells derived from normal subjects at 6 and 24 h (p = 0.02 and 0.01 respectively) The median values of IL-8 release from asthmatic cell cultures were 737.9 pg/ml for 6 hour and 1493.0 pg/ml for 24 hour stimulation (Figure 6 and 7). Pretreatment of cells with IFN-γ followed by toxin stimulation resulted in increased IL-8 release. Normal cells pre-treated with IFN-γ and then cultured with SEB at 1.0 μg/ml released 904.6 pg/ml IL-8 (median value). Cells from asthmatics treated in the same way produced significantly higher amounts of IL-8 – 1665.0 pg/ml (p = 0.01) (Figure 8 and 9).

#### TNF-α, RANTES and eotaxin release

Recent studies performed with *S.aures *added to airway epithelial cell culture have demonstrated robust response by secretion of proinflammatory cytokines including RANTES and eotaxin [21]. In our experiments TNF-α, RANTES and eotaxin were not detectable in cell culture supernatants collected at 6 and 24 h post stimulation with SEA or SEB. The detection limit for these assays was 2 pg/ml (data not shown).

### Spiking experiments

In order to ascertain that inability to detect RANTES, TNF-α and eotaxin was not due to fast degradation of cytokines in culture supernatant, spiking experiments were performed as described in Materials and Methods. Results obtained from these experiments showed that TNF-α was fully recoverable from all supernatants measured, whilst 90.8 ± 7.6% of IL-8, 74 ± 8.1 % of RANTES and 86.5 ± 7.7 % of Eotaxin was recoverable as measured by ELISA.

### Cell associated cytokines

In order to establish whether cytokines under study were stored intracellularly and failed to be secreted, we measured their concentration in cell lysates obtained from unstimulated cells and cells stimulated with enterotoxins. IL-8 was present in cell lysates from stimulated cells and was not detected in lysates from unstimulated cultures. TNF-α, RANTES and eotaxin were not detected in any of the patients' cell lysates (data not shown).

### HLA class II expression on nasal epithelial cells

Flow cytometric analysis of class II molecules expression revealed that only a small proportion of cells both from normal and asthmatic subjects were class II positive (healthy 2.5% ± 0.63, mean fluorescence (mf) 205 ± 40, asthmatics 2.03% ± 0.47, mf 193.8 ± 37.2). After stimulation with IFN-γ there was a small increase in percentage of HLA class II positive cells (healthy 10.2% ± 2.96, mf 1145 ± 301; asthmatics 11.76% ± 1.87, mf 1013 ± 189). No significant difference between cells from normal and asthmatic subjects was observed both under basal and stimulated conditions (p < 0.01). (Table 2)

**Table 2:** Flow cytometric analysis of HLA class II expression on nasal epithelial cells.

Human nasal epithelial cells	% positive	Mean fluorescence
Normal	2.5 ± 0.63	205 ± 40
Normal + IFN-γ	10.2 ± 2.69	1145 ± 301
Asthmatic	2.03 ± 0.47	193 ± 37
Asthmatic + IFN-γ	11.76 ± 1.87	1013 ± 189

Nasal epithelial cells were detached from culture dishes by means of nonenzymatic cell dissociation solution and were then stained with anti-HLA-DR, P, Q, FITC-conjugated monoclonal antibody. Membrane fluorescence was analysed by flow cytometry.

Involvement of MHC class II molecules in stimulation of HNEC by SEA and SEB.

Normal HNEC (n = 5) were pretreated for 24 h with 50 IU of IFN-γ/ml (or left untreated for control HNEC) and then were incubated with SEA or SEB (1 μg/ml) in the presence or absence of anti-MHC class II blocking antibody (50 μg/ml) as described in Material and Methods. Determination of IL-8 concentration in culture supernatants revealed that IL-8 secretion induced by SEA was inhibited by 40.5 ± 3.2 % in IFN-γ unstimulated cultures and by 70.2 ± 5.1 % IFN-γ treated cultures. For SEB stimulated cultures the obtained values showed 42.5 ± 3.2% and 68.9 ± 4.5%. These results indicate at least partial involvement of MHC class II molecules in SEA and SEB induced secretion of IL-8 from HNEC.

## Discussion

The main finding of this study is that SEA and SEA in non toxic concentrations directly stimulate nasal epithelial cells to produce IL-8 while they have no effect on TNF-α, RANTES and eotaxin production. These four cytokines have pivotal roles in the inflammatory response in the lung. IL-8 and eotaxin act as chemoattractants for neutrophils [25-27] and eosinophils [28] while RANTES is a chemoattractant for many inflammatory cells including eosinophils [29] and T lymphocytes [30]. TNF-α is a multifunctional cytokine which is known not only to stimulate most cell types to release other cytokines including GM-CSF [31] but is also known to stimulate the production of cytotoxic oxygen metabolites from eosinophils [32].

Since increased concentrations of IFN-γ are present in inflamed airways, the effect of preincubating cells with IFN-γ (50 IU/ml) prior to SAg stimulation was also investigated. IFN-γ has many proinflammatory effects and has been shown to play an important role in early childhood asthma through the upregulation of ICAM-1 [33] and the cellular receptor for TNF-α [34]. Other studies have demonstrated increased IFN-γ in BAL and blood from atopic asthmatics including acute severe asthmatics [35-37]. It is conceivable then that increased levels of IFN-γ in asthmatic airways result in upregulation of MHC class II expression seen in asthmatic airway epithelium [38]. In this study, cells from both subject groups were incubated with IFN-γ prior to toxin stimulation to investigate what effect if any there was on the release of IL-8.

Cells derived from normal subjects released IL-8 at 6 and 24 h in response to both SEA and SEB, with significantly increased responses in cells from asthmatic patients. Pretreatment of normal cells with IFN-γ followed by toxin stimulation resulted in increased IL-8 release. A similar trend was observed in IL-8 release from cells derived from asthmatic donors. IFN-γ pretreated cells derived from asthmatic subjects released increased levels of IL-8 in response to SEA (0.01 μg/ml) and SEB (1 μg/ml) at 6 h while at 24 h there was significant increase in IL-8 in response to both concentrations of SEA and to 1 μg/ml SEB.

The mechanism of how staphylococcal enterotoxins activate cells has been linked to their ability to crosslink the MHC class II molecules. The presence of HLA-DR antigens was demonstrated on IFN-γ treated airway epithelial cells but very little on unstimulated cells [39]. We also demonstrate that MHC class II antigen expression on HNEC can be upregulated by incubation with IFN-γ. The induced increase is however of moderate magnitude not exceeding 15% of cultured cells. No significant differences between normal and asthmatic HNEC were noted. Despite modest expression of MHC class II molecules on epithelial cell membrane, addition of blocking anti-HLA-DR antibody decreased IL-8 secretion in cultures of HNEC (40% reduction in unstimulated cells and 70.2% reduction in IFN-γ stimulated cells). These results indicate that enterotoxin binding to MHC class II receptors is at least partially responsible for the observed increase in IL-8 secretion.

Similar study using HNEC has recently been published showing that SEB induces proinflammatory cytokine secretion in vitro [40]. This study however did not investigate MHC class II expression and did not attempt to characterise the receptor responsible for SEB binding.

Some of the recent studies have shown that not all effects exerted by SAgs can be attributed to class II binding and crosslinking. Arad et al. showed that all superantigens have the ability to stimulate cross-immunity against each other through interaction of a dodecapeptide region of the molecules with a host cell receptor not involving MHC class II or T cell receptor [41]. Later Shupp and colleagues suggested that this receptor was important for superantigen transcytosis across mucosal surfaces [42] The staphylococcal enterotoxins which are members of the SAgs family have receptors on intestinal cells that lead to emesis and diarrhea associated with food poisoning; these biological effects are independent of superantigenicity [43,44]. While these results do not rule out direct effect of these toxins on intestinal epithelium they show that toxins can gain rapid access to the immune system. Further studies in this area using polarized nasal epithelial cells are clearly warranted. Finally, Paterson et al. showed that TSST-1 stimulates human vaginal epithelial cells to chemokine production via non MHC class II receptor [45]. It is therefore conceivable that interaction of SEA and SEB with nasal epithelial cells leading to IL-8 secretion described here can be mediated not only by MHC class II molecules but also by other yet undefined receptor and the effects described in this paper are due to enterotoxin binding to both MHC and non MHC receptors. It is also difficult to explain higher secretion of IL-8 from asthmatic HNEC in comparison to epithelial cells from normal cells since both groups expressed similar level of MHC class II molecules. We can only speculate that other, yet undefined receptor is responsible for this effect. This clearly requires further investigation.

Specific IgE to *S. aureus *SAgs is present in nasal polyp tissue, and levels correlate with markers of eosinophil activation and recruitment [46]. SEB selectively stimulates the production of interleukin-5 (IL-5) in patients with atopic eczema/dermatitis syndrome (AEDS) or allergic asthmatics but not in asymptomatic atopic or non-atopic individuals [6]. Given the central role of IL-5 in eosinophilia this provides further evidence that SEB may at least, play some role in allergic diseases such as AEDS and asthma. Further evidence that SAgs may play an important role in allergic diseases such as dermatitis, rhinitis and asthma comes from studies which report the prevalence of serum IgE antibodies to *S. aureus *enterotoxins. Sensitisation to *S. aureus *enterotoxins seems to be a factor in increasing serum eosinophil cationic protein (ECP) which is thought to be a reliable marker of clinical severity of allergic diseases including asthma and rhinitis [47].

As part of this study cell culture supernatants collected 6 and 24 h post SAg stimulation were analysed for TNF-α, RANTES and eotaxin. However, in all 20 subject samples these mediators were not detectable. *In vivo *administration of SEB to mice has been shown to trigger an inflammatory response characterised by mucosal and airway recruitment of lymphocytes, eosinophils and neutrophils together with elevated levels of IL-4 in BAL fluid [48]. The same study also demonstrated that SEB markedly enhances the frequency detection of TNF-α in BAL fluid [49]. However extrapolating results from animal studies and relating them to human studies must be done so with caution. It has been documented that murine cells are up to 1000 times less responsive to *S. aureus *enterotoxins than human cells [50].

Release of RANTES in response to SEB has been demonstrated in the human colonic T84 epithelial cell line [51]. In a model of human fibroblast-like synoviocytes, engagement of MHC class II molecules by SEA resulted in an increase in the mRNA level and protein synthesis of RANTES and IL-8 [52] while stimulation of human PBMCs to release RANTES in response to SEB has also been demonstrated [53].

In the human system of polarized bronchial epithelial cells a marked alteration in the transcriptional expression profile of epithelial cells in response to live *S. aureus *and soluble virulence factors was observed. These included pro-inflammatory cytokine release such as IL-1β, IL-8, eotaxin and RANTES [21,54]. It is possible that a mixture of soluble virulence factors induces vigorous proiflammatory cytokine response as a result of synergistic action of many bacterial products including exotoxins. Further studies using polarised HNEC should clarify the issue.

In conclusion this study indicates that SEA and SEB can induce an inflammatory response in human nasal epithelial cells. The responses to SEA and SEB are higher in asthmatic subjects and can be further elevated by preincubaion with IFN-γ. This would suggest that bacterial toxins such as SEA and SEB may play a role in the pathogenesis of asthma possibly via the recruitment of neutrophils into the asthmatic airway.

## Competing interests

The author(s) declare that they have no competing interests.

## Authors' contributions

GJOB carried out the cell culture experiments, analysis of cell culture supernatants and lysates and wrote the manuscript. GS introduced techniques used in the present study and carried out experiments related to MHC class II. GR recruited patients and performed nasal brushings, JSE ME and GS were involved in the design, supervision and writing of the manuscript. All authors have participated in the study design and evaluation, and have read, contributed and approved the manuscript.
